# Examining health care providers’ and middle-level managers’ readiness for change: a qualitative study

**DOI:** 10.1186/s12913-020-4897-0

**Published:** 2020-01-17

**Authors:** Tujuanna Austin, Samia Chreim, Agnes Grudniewicz

**Affiliations:** 1 Institute of Health Policy, Management, and Evaluation, University of Toronto, 155 College St, Toronto, M5T 3M6 Canada; 2 Telfer School of Management, University of Ottawa, 55 Laurier Ave E, Ottawa, K1N 6N5 Canada

**Keywords:** Readiness for change, Individual readiness for change, Primary care, Program integration, Healthcare management, Middle-level managers

## Abstract

**Background:**

Readiness is a critical precursor of successful change; it denotes whether those involved in the change are motivated and empowered to participate in the change. Research on readiness tends to focus on frontline providers or individuals in non-managerial positions and offers limited attention to individuals in middle management positions who are expected to lead frontline providers in change implementation. Yet middle-level managers are also recipients of changes that are planned and decreed by those in higher positions. This study sought to examine both frontline provider and middle manager readiness for change in the context of primary care program integration.

**Methods:**

Using a qualitative case study approach, we examined how frontline providers and middle managers experienced six readiness factors: discrepancy, appropriateness, valence, efficacy, fairness and trust in management. Data were collected through documents, meeting observation and semi-structured interviews with frontline providers and middle managers involved in the change.

**Results:**

The findings highlighted similarities and differences in readiness experiences of frontline providers and middle managers. Across both types of participants, we found that the notion of *valence* should be expanded to consider individuals’ evaluation of benefits to patients and the health system; *efficacy* applies to both content and process of change; fairness and trust in management findings require further exploration to determine their appropriateness to be incorporated in models of readiness for change; and *trust in management* (or lack of trust) has a cascading influence across the levels in the organization.

**Conclusions:**

Our study makes a contribution by nuancing and extending conceptualizations of individual readiness factors, and by highlighting the central role of middle manager readiness for change. Implications of the study include the need to consider readiness factors prior to the implementation of change and the importance of fostering readiness throughout all levels of the organization.

## Background

Health care systems around the world have been implementing changes to achieve better coordination of patient services and integration of programs to remove duplication of practitioner activities [1–3]. In Ontario, Canada, Health Links is one such program introduced by the Ontario Ministry of Health and Long-Term Care (hereafter “the Ministry”) to ensure coordinated, efficient, and effective care to patients with complex health and social care needs, typically patients with four or more complex or chronic conditions (*Ontario’s Action Plan for Health Care*, 2012; *Patient First: Action Plan for Health Care*, 2015). In this study, we focus on the integration of Health Links – a government mandated initiative that links patients with the various health care providers in their circle of care – with the Senior Care Program (name disguised for anonymity), a primary health service developed by and offered through community health centres. Whereas the objective of Health Links is to improve the quality of care for patients with complex conditions, the Senior Care Program serves to provide primary care, education, and social and practical supports to seniors to enable them to stay healthy and live more independently. The objective of the integration was to reduce duplication and ensure that patients in either Health Links or the Senior Care Program benefited from the tools and processes of both.

Implementing changes related to program integration can introduce challenges with bringing together divergent priorities, joining unique information processes and systems, removing overlap in various areas of operations, and restructuring roles of organizational members [4–7]. These changes can face resistance, making implementation of program integration difficult. In such contexts, it is helpful to consider readiness for change. Readiness for change is seen as a critical precursor of successful change [8–11]. Readiness captures whether those involved in the change are individually and/or collectively motivated and empowered to participate in the change [12]. Research shows that various factors can contribute to readiness such as leader characteristics, the change message, and individual characteristics and perceptions [8, 9, 12, 13].

Readiness can be studied at the organizational or individual level [11, 12]. Researchers have noted the importance of understanding the individual level, given that organizational change is a function of individual behaviour [14, 15]. Hence in this study, we focus on readiness for change from the individual perspective. However, the literature on readiness tends to focus on individuals in non-managerial positions, viewing these individuals as recipients of change and offering limited focus on those in middle management positions who are expected to lead the change. Yet middle-level managers are also recipients of changes that are planned and decreed by those in higher positions. These managers often tend to be viewed as change leaders and less so as individuals experiencing the change. When executives or policymakers design change projects, it is middle managers who operationalize these initiatives and act as intermediaries between top management and the frontline [16, 17]. The role of middle managers as recipients of change (particularly during mandated change), who also need to experience readiness, has not been emphasized in the literature. Herzig and Jimmieson state that “While the facilitators and barriers to change have been previously explored…for employees, particularly in relation to how employees cope and adjust to organizational change, there is little research about how these processes operate for middle managers” [18]. While there is strong recognition that “studying middle managers is imperative” [19] because of their unique role in facilitating health care change acceptance and bridging between senior leaders and frontline staff [19, 20], researchers have noted that middle managers have not received the attention they deserve [19, 21]. Hence, our study focuses on both middle level managers’ and frontline providers’ readiness for change in the context of program integration.

In the extant literature, there is not a single, universally accepted model of individual readiness for organizational change. However, there are five elements of readiness that we have collated from various authors’ work [8, 9, 22–25]: *discrepancy, appropriateness, valence, self-efficacy* and *fairness*. We focus on these five elements based on our reading of the literature, but it is possible that other researchers considering readiness may include a more limited set of factors or a similar list with different terminology, which points to the lack of a definitive list. We describe each of these five elements briefly as we will use them in our analysis of frontline providers’ and middle managers’ readiness for change. *Discrepancy* refers to the perception of the need for change, or a perception of imbalance between the current state and the desired state [9, 26]. Individuals look for indications that the future state, to be achieved through a given change, will be preferable to the current state [9, 27, 28]. The perception of discrepancy creates a motivation for change through the generation of dissatisfaction with the status quo [29]. To be motivated to change, an individual must also believe in the *appropriateness* of the given change, i.e. that the proposed change is an adequte response to the discrepancy [8, 9, 14]. Appropriateness is distinct from discrepancy since individuals may believe that a change is needed, but may disagree with the proposed course of action [30]. Having a clear rationale for the proposed change, instead of another change or no change at all enhances the sense of appropriateness [31].

*Valence* is defined as an individual’s assessment of how the change can be beneficial and whether it is worthwhile [9, 11, 32]. The greater an individual’s perception of the benefit of the change, the more likely they are to want to participate in the change. In line with the theory of motivation, individuals will expend effort on activities for which there are perceived rewards [33]. *Self-efficacy* is an individual’s confidence in their ability to participate in and implement change successfully [9, 22, 34, 35]. Bandura (1982) notes that individuals will avoid activities perceived to be outside of their capabilities, but will participate in those that they judge to be capable of. Training surrounding the content of the change facilitates self-efficacy since it enhances individuals’ knowledge and skills to perform the new tasks associated with the change [12, 34, 35].

*Fairness* is an element of readiness that some authors include and others omit from their frameworks [9, 12]. Individuals involved in fair change initiatives are more likely to display commitment to the change and to better adjust to adversities of the change [25]. There are two forms of fairness: procedural and distributive. Procedural fairness is perceived fairness of the process of the change [24] and relates to how decisions on the change are made. Giving voice to individuals affected by the change so they are able to express their views and interests enhances the sense of procedural fairness [24]. Distributive fairness is perceived fairness of the outcome of the change [24, 25, 36]. Distributive fairness is most often associated with an individual’s perceived equity in resource allocation [24]. Resources are not limited to tangible items such as financial resources but may include intangible elements such as power, authority, and responsibility.

Our objectives in this study are to understand individual readiness for change of both middle level managers and frontline providers in the context of program integration, and to explore the differences in readiness between these two levels in the organization. To achieve these objectives, we conducted a qualitative study in order to gain an in-depth understanding of individuals’ own perspectives of their readiness for change. This is in contrast to much of the literature on readiness, which tends to adopt a quantitative approach with use of various readiness measurement instruments [10]. Although quantitative studies have offered valuable insights, they fall short of delving into the experience of participants as recounted in their own words.

## Methods

### Study context

The case that is the focus of this study is the integration of Health Links with the Senior Care Program. The integration was in its early stage at the time of data collection in 2017–2018 and had started to be rolled out in some but not all sites we studied. Table 1 presents a brief description of the two programs. The Senior Care Program is a well-established service delivery program offered to high-risk seniors living at home. The program had been running for over eight years at the time of the study. As part of the program, registered nurses and community health workers perform in-home assessments, collaborate with seniors’ primary care providers, provide education, social and practical supports to seniors, and assist with case management and system navigation. The Senior Care Program is led through a Lead Site, and operates in several additional satellite sites. The Lead Site provides direction and financial support to the satellite sites, who also each have their own management teams.

**Table 1:** Description of the Senior Care Program and Health Links

	The Senior Care Program	Health Links
Description	An interdisciplinary primary health service delivery program offered through 10 Community Health Centres (CHCs) in Ontario	An approach to coordinating care that links patients, their health care providers, and their caregivers together through a coordinated care plan that documents the roles, responsibilities and contact information for each individual
Mandate Structure	A program that reports to the Regional Authority, is directed by the Lead Site, and is composed of various satellite sites	A province-wide initiative mandated by the Ministry
Objective	Provide community-based services to frail seniors (and their caregivers) to enable them to stay healthy, live more independently, and avoid unnecessary use of costly emergency rooms in hospitals	Improve the quality of care and the health care experience for those who use the health system the most, and coordinate care for the top 5% of health care system users
Target Population	Frail seniors (aged 65+)	Patients of any age with complex conditions
Objective of the integration	Enhance coordination of care and health service delivery for seniors with complex conditions	

Health Links was first introduced by the Ministry in 2012 to ensure coordinated, efficient, and effective care to patients with complex health and social care needs. The initiative started with early adopters and used a staggered approach to the implementation of new networks, resulting in 82 approved Health Links networks at the time of writing, all in different stages of implementation [37]. It targets the top 5% of health care system users, typically patients with four or more complex or chronic conditions (Health Links, 2018). A central element in Health Links is the *coordinated care plan* – a document that details all actors and their roles in a patient’s circle of care, including caregivers, health care providers and external social care agencies (e.g. Meals on Wheels). Following the Ministry’s goals, the Regional Authority (the administrative level of government responsible for the local delivery of health services) had directed the Senior Care Program to integrate Health Links in its operations. Thus, the formal chain of authority for this change from top to bottom was as follows: the Ministry ➔ the Regional Authority ➔ the Lead Site management ➔ local (satellite) sites management ➔ frontline providers. In this chain, we considered the managers at the Lead Site and at the satellite sites to be middle managers. It should be noted that this chain of authority was not always followed; sometimes the Ministry would communicate with Lead sites directly rather than through the Regional Authority.

Integration of Health Links with the Senior Care Program began with training at some sites on the coordinated care plan and the introduction of a new medical chart documentation system where the coordinated care plans would be stored and updated. The various site managers were responsible for leading the integration of Health Links at their own sites in collaboration with leadership from the Lead Site. Several sites engaged “champions” or individuals who had previous experience with the Health Links approach to help with the implementation. Frontline providers, most of whom were Senior Care Program workers, were responsible for completing and managing the Health Links coordinated care plans for eligible patients, and organizing care conferences (meetings with the individuals and agencies involved in the circle of care). Implementation of Health Links was ongoing at the time of writing this paper.

### Approach and rationale

We used a qualitative case study methodology. Researchers have noted the usefulness of qualitative research in informing health management through the detailed investigation of what individuals do and why they do it [38]. Qualitative research is also valuable for understanding participants’ frames of reference [39], which is particularly relevant in a study of individual readiness for change. The case study is a research design that involves in-depth data collection from different sources with the objective of understanding the dynamics and complexities present within the case [40].

### Data sources and collection

The study received ethical approval from the University Research Ethics Board. Permission to conduct the study was granted by management at the Lead Site. Written informed consent was obtained from participants before beginning interviews and meeting observation. One member of the research team (AG) had a pre-existing relationship with one of the Lead Site managers from a previous research project. In advance of data collection, members of the research team met this manager to discuss the project.

Data sources consisted of semi-structured individual interviews, non-participant meeting observation of a three-hour Senior Care Program regional meeting, and written documents (meeting agendas, meeting notes and training documents). The primary source of data were the interviews and meeting observation, while the documents were intended to provide contextual information on the integration. Interview participants were identified through both purposeful and convenience sampling approaches. Purposeful sampling targeted: 1) individuals involved in managing or leading the Senior Care Program at the Lead and satellite sites; and 2) individuals providing care to patients as part of the Senior Care Program and/or involved in care coordination as part of the Health Links approach. Once interviews were conducted with managers at the Lead Site, other participants involved in the integration were invited to participate in the study. Lead Site managers were asked to share the recruitment text with participants involved in or affected by the integration, and to tell members that those interested in participating in the study should contact the researchers directly. Thus, all individuals (managers and frontline providers) in the Lead Site and in satellite sites involved in the integration of Health Links and the Senior Care Program were invited to participate in the study (*n* = 36). Of these individuals, 18 managers and employees agreed to participate in the interviews. There were no individuals who held dual roles as managers or care providers nor were there any individuals deemed ineligible. Participants were made aware of the study objectives and the research team’s interest in the study topic. Interviews lasted between 60 and 90 min and were conducted between March 2017 and February 2018 in private offices at the community health centres. Interviews were audio recorded and transcribed verbatim and field notes were made during and after the interviews. Table 2 presents an overview of the study participants.

**Table 2:** Study participants

Position	Number of participants
Managers	5 (3 managers in the lead site and 2 managers in satellite sites)
Frontline providers (including nurses and community health workers)	13 (3 providers in the lead site and 10 providers in satellite sites)

A semi-structured interview guide was used with questions pertaining to roles, context, readiness for change, leadership, and obstacles to/enablers of the change. (Please refer to Additional file 1 for the interview guide). All interviews were conducted by the first author (TA), a health services researcher with training in qualitative methods. A semi-annual Senior Care Program regional meeting was held in January 2018 for managers and health care providers from all sites. One member of the research team (TA) was present at this meeting as a non-participant observer. Semi-annual meetings are intended to provide updates and information to all Senior Care Program sites. The meeting in January 2018 provided staff with information on the integration and a platform to voice concerns to Lead Site management. The research team member took notes on the topics discussed, individual reactions to these topics, and common questions raised by the audience. These notes were used as a primary source of data. Participants were also asked to share any documents that pertained to the integration. We received nine documents that contained information on training conducted, meeting notes from managerial meetings, and action items for discussion with the Regional Authority and the Ministry.

### Data analysis

Interviews and meeting observations were analyzed according to procedures outlined by Miles et al. [41]. Atlas.ti software was used to facilitate coding. Coding began with a provisional start list of codes [41] informed by the literature (e.g. the five readiness factors we outlined earlier) and the interview questions. The analytic coding tree began with this deductively derived start list of codes. We remained open to themes in the data that were not captured by our initial codes. For example, in addressing their readiness for change, participants referred extensively to their views of managers, and whether they believed the managers to be trustworthy and capable of providing valuable information on the change and managing the change process. Therefore, we added the inductive theme of “trust in management” to the list of readiness factors, defining it as the belief that those managing the change are capable of providing support and implementing the change.

As the analysis progressed, nuances in the data started to emerge. This led to the creation of a set of pattern codes or themes [41] along with subthemes. For example, an initial code had been created for Valence - one of the readiness factors in the literature. Over time, it became clear that Valence did not refer only to benefits for the individual, but also to those for the patients and the health system. Thus, Valence was designated as a pattern code or theme, with subcodes related to various areas of benefit as recounted by the participants. Development of the code list continued in this iterative manner. All interviews were recoded with the final code list. Please refer to Table 3 for the coding tree.

**Table 3:** Coding Tree

Theme	Sub-Theme	Example Quote
Discrepancy	Current inadequate patient services	We have our problems, as all places do, this new approach could help to fix some of the gaps
	Need for coordination	[We need to] get everybody talking, everybody who’s involved, and make sure the information doesn’t slip through the cracks.
Appropriateness	Enhanced patient services	I think the more you know about [Health Links] the more you understand how it works… and why it’s a good fit
	Integration enhances partner relationships	You seek a clear difference in the way we work with our partner agencies in this approach…sure we’re all still figuring it out but it has clearly enhanced our communications with them
Valence	Personal valence	I hope that the integration will reduce my caseload
	Patient valence	A lot of my clients appreciate having all their contact laid out for them
	Health system valence	This really has the potential to change the system, but only if it’s done right
Self-Efficacy	Formal training	There’s been some sessions organized by [Regional Authority] but nothing particularly helpful
	Informal training	There was a lot of out of the box training that wasn’t organized by [Lead Site] or the Ministry
	Champions	I think I’m sort of a champion for the approach, I’ve done some additional training and people come to me when they have questions… I’ve been told I’ve been helpful
	Change management capability	We may be marginally trained in Health Links, but we are definitely not trained in how to change
Fairness	Procedural fairness	[Name of site] had meetings and meetings with [name of manager] to discuss Health Links and we literally had none
	Outcome fairness	There’s no way this is sustainable without more staff. We don’t have as many people as [other satellite site] –
Trust in management	Perception of middle managers	I have my own feelings about management that…shape my feeling of Health Links
	Perception of Regional Authority	[Regional Authority] doesn’t exactly provide us with clear ways forward.

Initial coding was performed by TA, with revision and confirmation of coding performed by SC. TA and SC worked together on analyzing the primary data (interview transcripts and meeting observation notes). The third researcher (AG) reviewed extensive coded data and provided feedback on the analysis.

The analytical process described above involved using the techniques of *looking at the big picture* and *telescoping* [42]. Looking at the big picture refers to the task of examining all elements of a concept and showing their relationships, and telescoping refers to moving from broader to detailed perspectives [42]. To achieve trustworthiness of the findings, we triangulated interview data with the meeting observation material. Triangulation was also achieved through participation of multiple researchers in the analysis process. Additionally, by providing rich description of the case and extensive quotes from the data, we allow readers to form their own judgements about the analysis [41]. A report of the study findings was shared with the two managers whom the researchers had initially. Their feedback was sought to ensure that the analysis captured participant perspectives. Their comments indicated that the report “is a true reflection” of the dynamics that were present at the sites at the time of the study.

## Results

**Fig 1.**
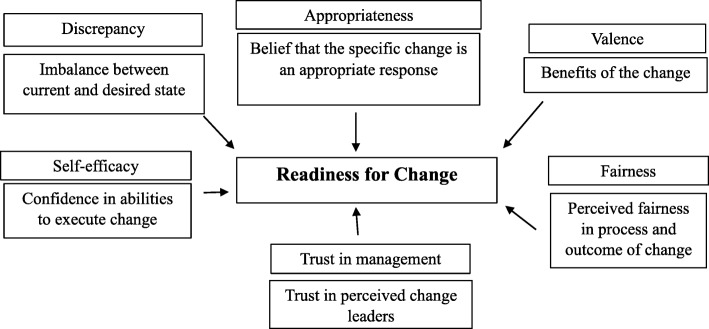
Readiness factors

In total, 18 of 36 eligible managers and frontline employees participated in the study. We summarized the readiness factors based on our findings in Fig. 1. The data showed variations in the readiness factors that contributed to some frontline providers being ‘more ready’ for the Health Links and Senior Care Program integration than others. There were also similarities and differences between frontline providers and middle managers. Table 4 provides an overview of the readiness factors and how they manifested.

**Table 4:** Factors enhancing and hindering readiness for change

	Factors that Enhance Readiness	Factors that Hinder Readiness	Differences Between Managers and Health care Providers
Discrepancy	-Encountering challenges with the status quo of health service delivery with the Senior Care Program	-Limited awareness of problems associated with the current state	-Discrepancy experienced similarly by frontline providers and middle managers
Appropriate-ness	-Detailed knowledge about the Health Links approach	-Perception that Health Links is a duplication of the Senior Care Program -Poor rapport with Senior Care Program management at the Lead Site	-Newer employees and middle managers were more likely to view the integration as appropriate when compared to other providers
Valence	-Seeing value to Health Links at the system and the patient level	-Perception of increased workload with the implementation of Health Links (e.g., double documentation)	- Frontline providers mostly discussed benefits of the integration at the patient level and managers discussed the benefits of the integration at the health system level
Self-Efficacy	-Informal training (e.g. job shadowing) -Learning by doing	-Formal training not comprehensive enough (focused on limited aspects of Health Links) -Lack of training on change management	-Self efficacy experienced similarly by frontline providers and middle managers -Middle managers emphasized the need for training on change process and management
Fairness	-Working at sites with more administrative support and more opportunities for training	-Not being consulted on the change -Working at sites with minimal opportunities for training and no administrative support	-Lack of procedural and distributive fairness experienced similarly by frontline providers and managers
Trust in management	-Clear communication from local managers -Local managers’ ability to support and provide solutions	-Limited or unclear communication from managers at higher levels -Leadership ambiguity -Not knowing who to turn to for information or support	-Trust in management experienced similarly by frontline providers and middle managers. -Lead Site managers themselves viewed contact with and information from their superiors as lacking

Although these factors are shown as distinct from each other, it should be noted that they are not mutually exclusive, and that there might be areas of overlap between them.

***Discrepancy***, participants’ perceptions of a gap between the current state and the desired state was experienced similarly by both frontline providers and middle managers. Participants – regardless of their role – noted that the prevailing organization of health service delivery and the current operations of the Senior Care Program (the status quo) were not optimized to provide care to patients who had multiple chronic disorders and required continuous care. Most participants described the shortcomings of the current state, focusing on the challenges of communication between health care providers and of information sharing and coordination among agencies. These factors contributed to a perception of discrepancy.

*So let’s say I have a client and I go to the specialist with him and he is asked all these questions that he’s already been asked beforehand, or you go to the family doctor and he didn’t get the report or I didn’t know that [Geriatric Society] was involved because the person didn’t remember. You’re trying to work together, but if you don’t have all the pieces of information, sometimes you do the same work as someone else so you’re duplicating the service. It’s a lot of work now but if we’re all working together and everyone is informed, then ideally it’s going to be a lot less work later.* – Frontline provider.

*It’s because of poor communication among agencies. When we see these people, sometimes they have multiple services and it’s just a lack of coordination as well. Nobody knows who’s doing what.* – Manager, Satellite Site.

***Appropriateness,*** the second readiness factor, denotes the perception that the specific change is the most appropriate course of action. In this case, although participants recognized the need for a change (i.e., perceived there to be discrepancy), not all of these individuals believed that integrating the Health Links approach with the Senior Care Program was the most appropriate course of action.

Middle managers, champions, and newer employees of the Senior Care Program tended to say they viewed the integration as appropriate, and Health Links as complementary to the Senior Care Program. Managers were more closely linked to the Regional Authority, which strongly advocated for the integration. More importantly, as ambassadors for the change, managers and champions were responsible for leading the implementation of Health Links in their sites and likely would have bought in to the integration. Alternatively, managers may project the notion that the integration is the appropriate course of action in order to bolster employee morale and create a sense of appropriateness among employees given that the integration was mandated and the change was to occur regardless of individual opinions of the change. When “selling” the appropriateness of the Health Links–Senior Care Program integration, managers and champions emphasized the enhanced relationships with partner agencies.

*Some Senior Care Program people tend to work in silos and just do outreach to primary care providers, and just decide to do everything themselves. If we use the Health Links approach, at least we get more people connected with our client. I think it is a good approach and people should give it a try.* –Frontline provider (champion).

Health care providers who noted the implementation of Health Links to be an appropriate course of action also appeared to have a better rapport with both local and Lead Site Senior Care Program managers. For new employees, the perception of appropriateness was linked to their joining the Senior Care Program during a time of change, and the messaging that they received from management and change champions during this time. Newer employees were generally more open to change as they did not operate under the older way of providing services.

*We get a lot of information when we do our home visits and we go back and put it in our charting system, but not everybody has access to it. Health Links is really trying to make sure that the care plan, once it’s completed, is going to be out to as many people as possible and get the integration.* – Frontline provider (working with Senior Care Program for 3 months).

Individuals who perceived the integration of Health Links to be an inappropriate course of action believed that Health Links did not add any value to the work being done through the Senior Care Program. While these individuals identified discrepancies with the current state, they did not believe that integration of Health Links would resolve these discrepancies.

*What I’m hearing is that we’re doing it to lessen our workload… so I can do this whole document up and it says who’s part of my health care team and I know that we’re saying okay, if you’re from Meals on Wheels I want you to have a certain responsibility to make sure you get the meals to the client, but we’re already doing that as Senior Care Program staff, like I’m already coordinating that circle.* – Frontline provider.

Individuals who held these opinions tended to more frequently mention poor communication from management about the change, which led to poor understanding of the Health Links approach. Individuals who did not perceive Health Links to be appropriate also expressed some confusion about how Health Links differed from the Senior Care Program.

***Valence*** refers to an individual’s assessment of whether or not the proposed change is beneficial and worthwhile. Participants discussed valence on three levels: the individual/personal level, the patient level, and the health system level. Frontline providers and middle managers who spoke about benefits of the integration on more than one level were more likely to perceive the change as valuable. It is interesting to note that frontline providers mostly discussed benefits of the integration to their patients while the managers tended to focus on the benefits of the change to the health system (e.g., reduced resource consumption).

*Especially in the senior population when you start taking away their driver’s licence, taking away some of their rights to decide whether to stay home or go into residences, people tend to be a lot more resistant. But for us to be presenting them with all the facts and all the options, they’re able to make these important decisions about their own health and they feel like they’re being heard.* – Frontline provider.

*I’ve been kicking the can a long, long time…and this is the first time I believe there’s ever been a program that can make a systems change.* – Manager, Lead Site.

Among the frontline providers who did not perceive the integration to be valuable, was the view that the Senior Care Program was already doing the work of Health Links and that the integration would bring additional workload. Hence, these individuals viewed the change as having negative valence. Another factor contributing to lower perceptions of valence from the frontline was poor communication from management on the objectives of the integration, which resulted in some individuals not clearly understanding the entirety of the change, in turn creating a perception of low value of the change.

*I: For planning of Health Links integration with the Senior Care Program, were there any objectives that you wanted to reach?*

*P4: If there were objectives…, they were not shared with us.* – Frontline provider.

***Self-efficacy*** refers to an individual’s belief in their ability to undertake activities associated with a change. Strength of self-efficacy varied across participants, whose accounts showed one or more of the following experiences: feeling efficacious, feeling trained but not equipped with the tools/resources necessary to actively undertake the change, having a sense of “learning as you go”, and having a sense of confusion and lack of direction. Some people exhibited aspects of higher and lower efficacy depending on the activity they were talking about. Both frontline providers and middle managers exhibited a variety of experiences of efficacy.

When discussing some of the formal training organized by the Lead Site, individuals stated that they did not find this training useful in helping them to feel competent in their abilities to undertake activities associated with the integration. Formal training varied across the sites, but in most sites, it primarily consisted of training on the use of the new information technology system (where the coordinated care plans were stored) and on how to complete those plans. There was no formal training on other aspects of the change such as organizing care conferences involving various practitioners and care agencies, and gaining patient buy-in on the Health Links approach. The general view was that the formal training was a good introduction to the Health Links approach but was not useful for assisting providers to practically implement the approach.

*Honestly, [training] was a waste of my time… Like it’s all straightforward…, the two hour training…our training we did was basically going through the coordinated care plan* – Frontline provider.

A frontline provider noted that the training created more questions than it answered:

*It was jam packed full of information, I think it was a two day training I had, but I still had a lot more questions than I had training.* – Frontline provider.

In contrast to formal training, informal training – i.e., learning from other individuals who had had successes using the Health Links approach and “wading through the mud together,” as an interviewee described it – tended to enhance participants’ sense of efficacy.

*In my first case conference, I had a care coordinator sit in with me and she helped facilitate that, but she also does her job in geriatric mental health so I was able to see her wear her two hats in the meeting … It was interesting, just to get everybody together*. – Frontline provider.

Participants mentioned “jumping in with both feet” or learning as you go as a way to enhance self-efficacy. This was consistently noted by both frontline providers and managers. In the ambiguous context of this particular change, it was not surprising that individuals’ belief in their abilities to implement the change increased as they participated in the change.

*I don’t know how you get ready because as messy as it’s been, until you jump into something and you get into the messiness of it, I don’t know how we would’ve gotten there. –* Manager, Satellite Site.

Interestingly, managers noted the importance of feeling efficacious in relation to the process of change management (which was not achieved in this case), and not only in relation to how to become proficient in the Health Links approach.

*This is a change process and I think there almost should’ve been training around change management and how to support an organization around that* versus *just Health Links. This is a potential system overhaul for all of us, so that would have been helpful.* – Manager, Satellite Site.

***Fairness*** refers to the perceived justness and equitableness of the process and outcome of the change. Lack of procedural fairness was mentioned by both frontline providers and middle managers, who stated they were not consulted on matters related to the change that directly impacted their work:

*When Health Links was introduced, we were not involved in the planning at all. It was more of a ‘we’re doing Health Links’ and that was the end of the conversation. –* Frontline provider.

*There was a lot of talk about Health Links coming, but I was not invited to be involved in any of the planning … There was a lot of stuff I had questions about and [their expectations for me] weren’t clear so that made things even more confusing. –* Frontline provider.

Frontline providers and middle managers also mentioned lack of outcome or distributive fairness, typically in reference to disparities in training and administrative resource allocations. Perceptions of training disparities were associated with the amount and quality of training offered. At the regional meeting that we observed, a frontline provider said, “*I know that some sites are receiving training that we didn’t even know was available to us. We’re supposed to be in this together, but we’re not. It’s hard to go through the fog when we’ve got mud in our eyes*.” Another frontline provider stated in interview that the individuals who were supposed to provide training “*went around and visited all the sites to provide a bit of training on Health Links. They kept rescheduling our training and when they did come in, it was not even 15 minutes, when I know they spent a lot more time at the Lead Site.*”

Consistently with disparities in training, one middle manager told us that she participated in a lengthy Health Links training program, while other managers did not:

*The Business School was paid by the [Regional Authority] to deliver a course around Health Links - what’s collaborative, what leadership looks like and skill building exercises. Within that, the conversations were always about what does Health Links look like… At the end of the 3 months, all of us had a much clearer vision about Health Links*. – Manager, Satellite Site.

There were also perceptions of administrative support disparities - specifically uneven availability of project management and administrative support - that contributed to a sense of lack of fairness among some individuals. At the regional meeting, this issue became clearly evident when it was disclosed that some sites received complete administrative support while others received part time administrative support or none at all. Only some sites had access to Health Links coordinators who took on the task of care coordination, which created workload disparities:

*We have no admin support. We have to do all of our [document work] ourselves, but other people I talk to don’t have to do that.* – Frontline provider.

***Trust in management*** refers to the belief that those managing the change are capable of providing support and implementing the change. This factor emerged from the data and, interestingly, was mentioned by frontline providers and managers in the satellite and lead sites. While many frontline providers tended to view the management team at the Lead Site as unhelpful in terms of providing information and direction, they frequently shared that they trusted local managers:

*We have a great director who embraces change or anything that enhances patients’ health. And there’s tremendous support and go-to people here that if you have a question they’re here to assist you.* – Frontline provider.

An impediment to trust in higher levels of management was ambiguity in terms of who was exercising leadership. Both frontline providers and middle managers referred to leadership ambiguity as a challenge. The leadership structure of the integration included various actors with different and sometimes unclear roles. In some instances, the Regional Authority provided directives to the Lead Site, which then disseminated information to the satellite sites, and in other instances, the Regional Authority provided information to all sites directly. In addition, management in the Lead Site indicated that they were not given sufficient information to relay to other sites, and that they were in the dark regarding central issues related to the integration:

*I think directions haven’t been clear in the last two years. I am looking back at notes and questions we had a year ago at meetings, and they are still the same questions [today]. –* Manager, Lead Site.

Leadership ambiguity and lack of understanding of the roles played by different managers was a common theme:

*…I know a lot of people who don’t know exactly who to turn to…* – Frontline provider.

There was much confusion about administrative processes pertinent to the integration. Frontline providers and middle managers alike complained about lack of clear directives for their role in the integration. Several participants mentioned that there were no clear directives with respect to double documentation (having to chart in both Senior Care Program and Health Links systems) and information sharing with community partners who had not yet adopted the Health Links approach – all of which contributed to a sense of lack of support from some levels of management.

## Discussion

This study sought to examine readiness for change from an individual (frontline provider and middle manager) perspective during the early stages of integrating a new initiative with an existing program of care. In this section, we discuss our findings on six central readiness factors and on the middle managers’ experience of readiness.

### Readiness factors

The findings support the notion that dissatisfaction with the current state (discrepancy) is an important element of readiness [9, 28, 43]. Both frontline providers and middle managers highlighted dissatisfaction with the current state, noting that the Senior Care Program was not optimized for patients with complex care needs. The literature notes that appropriateness is complementary to discrepancy since a perception that the proposed change is the most appropriate course of action cannot exist without a perception of dissatisfaction with the status quo [9]. The findings show that some individuals found the change appropriate, while others did not. A key aspect of appropriateness is that change recipients understand the rationale behind the proposed change, which was not clearly communicated in the case we studied.

While the literature on valence tends to focus on an individual’s perception of how the change benefits them [9, 11, 12, 32], our study contributes to this literature by showing that valence can extend beyond personal benefits. Our findings clearly show that valence included consideration of patient and health system benefits - areas of valence that are not considered in depth in the literature on individual readiness. In the health care context, providers attach high significance to the benefits that accrue to their patients, and managers attach high significance to systems benefits due to suggested changes. Frontline providers and managers who noted more benefits were more accepting of the change. Valence is enhanced when the change is seen as having multiple benefits.

Research has shown that the higher an individual’s self-efficacy, the more likely they are to participate in a change [12, 22, 32, 34, 35]. There is emphasis in the literature that change might require new work skills, thus the importance of training is emphasized [22, 32, 34, 35]. This is consistent with the results of this study, but the study also helps extend understanding by highlighting the significant benefits of informal training (e.g., job shadowing) and learning by doing. As a participant stated, “wading through the mud together” was in effect a way that participants were able to learn. This shows the importance of experiential learning. In our study, participants referred to informal training, observation, and learning by doing as better sources of learning than the formal training that was provided. In addition, the findings show the importance of providing training not only on the content of the change (e.g., new skills required to use a new information system), but also on the process of change. This aspect of self-efficacy is particularly important for those tasked with managing the change (e.g., middle managers).

Fairness is one of the factors that is less studied in research on readiness for change. Several models of individual readiness that we reviewed do not include this notion [8, 9, 11–13]. This study highlights the importance of fairness and presents concrete examples. Individuals reduce their commitment to projects they perceive to be unfair. In our study, a sense of lack of fairness appeared to arouse emotional reactions from participants. While it is possible that the distribution of resources (e.g., training time, administrative support) in our study was uneven due to differences in Senior Care Program sites, this does not seem to have been well communicated. Research suggests that perceptions of fairness depend on the information available during the change [44]. The findings of this study point to opportunities for building research on the notion of fairness as a readiness factor.

Trust (and lack of trust) in management was an important theme in our participants’ accounts. The literature indicates that trust “is about the relationship between those who are being asked to participate in the change, to make a personal transition, and those who are doing the asking, whether they are senior managers or local managers” [45]. While Armenakis & Harris (2002) and Rafferty et al. (2013) have referred to “principal support from management” as a factor that facilitates change, we view the notion of “trust in management” as a better descriptor of individual readiness since it refers to a belief held by the recipient of change. Trust in management involves believing that change agents have the competence to manage the change successfully – that they are capable of providing tangible support in the form of resources and information and of engaging in open communication [44, 45]. Frontline providers expressed trust in their local managers, but had generally negative views of the more removed levels of management. Participants in the satellite sites were generally critical of the role of managers in the Lead Site and beyond. Interestingly, managers in the Lead Site themselves also had experiences of lack of support from the Regional Authority, indicating that lack of information and resources cascaded down from the senior levels to the frontline level. This cascading effect points to the challenging position that middle managers occupy: if they lack information on the change that was conceptualized at higher levels, they will likely experience difficulties in making sense of the change for themselves and for others who report to them. Future research would benefit from considering in more depth how ambivalences experienced by managers affect employees’ readiness for change.

This paper also brings to light the interesting discussion on when readiness for change occurs and when it should be studied. The literature typically portrays readiness as a precursor to the behaviors that support change [9, 12], but two important elements should be considered. First, change in organizations seldom proceeds in two distinct phases of planning (which is when readiness would ideally be created) and implementation. Rather, some implementation may be under way before all planning has been completed. This is often the case in Ministry-led initiatives that rely on iterative development and implementation strategies that start with a more flexible and adaptable approach [46]. Second, readiness might develop as one engages in some change behaviors. As some of the participants stated, “learning by doing” is important, and part of readiness involves “jump[ing into the change] with both feet”. Hence it is important that future research consider the timing of readiness, and whether it is a single occurrence or an evolving phenomenon.

### Middle managers’ readiness for change

The success of organizational change greatly depends on how the change process is managed, and how well managers prepare employees to adopt and embrace the change [8, 28, 47]. There were several layers of management involved in the integration of Health Links and the Senior Care Program (the Ministry, the Regional Authority, the Lead Site managers, and the local site managers) whose roles were not clearly delineated and understood. This posed a challenge to the implementation of the change.

Managers at the lead and satellite sites were in a middle management position. In this position, managers are both recipients and agents of the change [48]. Middle managers play key roles in interpreting, communicating, and implementing change as they work towards unrolling change initiatives in line with executive direction [16, 17, 27]. As the intermediaries between upper management and the frontline, middle managers as change leaders face immense pressure to unfold the change in accordance with direction from upper management while promoting the change and creating readiness among their employees [49]. This can be challenging because change leaders need to “own” the change in order to communicate it effectively, but if it is someone else’s idea, “communication can be weak” [50].

In our study, this dual role proved challenging for the middle managers. As recipients of change, these managers received information about the integration from senior management (i.e., the Regional Authority) and were tasked with implementing the change, irrespective of their individual views or level of readiness. Yet, there were unclear expectations from senior management. As agents of change, middle managers were responsible for communicating information about rationale, objectives and process of change to the frontline. Frontline employees relied on them, often without recognizing that these managers were also recipients of change, who often lacked adequate information and resources to enable change. Middle management positions are key in facilitating change implementation and creating readiness for change [48], and deserve more research attention.

## Conclusion

The current study contributes to the literature by extending understanding of readiness factors and by highlighting the importance of considering middle management readiness for change. With respect to the readiness factors, the study contributes by a) providing evidence that the notion of valence should be expanded to consider how individuals evaluate benefits of the change to themselves, to their patients and to the health system; b) showing that fairness and trust in manager findings require further exploration to determine their appropriateness to be incorporated in models of readiness for change; and c) indicating that self-efficacy applies to both content and process of change and that efficacy can be achieved through various means such as observation and experimentation, and not only through formal, often didactic training approaches The study also contributes by emphasizing that readiness for change should be considered not only at the individual frontline employee level, but also at the individual manager level.

By using a qualitative method of inquiry, this research was able to provide a contextualized examination of individuals’ experiences of readiness for change factors. While qualitative research is not generalizable [41], the findings of this study would be of value to those in health care settings undergoing early stage program integration and other types of changes. It is conceivable that other individuals facing changes in organizations would experience readiness based on whether discrepancy, appropriateness, valence, efficacy, fairness and trust are attained. In addition, this study took place in the context of a change mandated by senior management that is removed from the day-to-day operationalization and implementation of the change. It is plausible that the challenges observed in the study would be similar in other situations where middle managers lack information and resources related to the change.

This study has limitations. It did not include individuals from top management or policy positions (e.g., the Ministry or the Regional Authority) which limited exploration of readiness to managers in middle-level positions. Future research would benefit from including the perspectives of additional levels of management in order to reach more comprehensive understanding of the dynamics at play. Another limitation is that the study did not attempt to provide a comprehensive framework for the study of individual reactions to change; rather the focus was on a set of six elements that are important in understanding individual readiness. Future research may investigate not only the perceptions and cognitions of individuals, but also their emotions towards the change [8]. Along these lines, our findings suggest that overall readiness is determined by discrepancy, appropriateness, valence, efficacy, fairness and trust, but more research is required to establish the extent to which each of these elements contributes to overall readiness.

From a practice standpoint, this study suggests that a thorough understanding of readiness is useful for health care managers. Elements of readiness should be considered early in the planning phase of a change project. Specifically, this study can be used to assist managers in shaping the communication to frontline employees when planning and implementing organizational change initiatives. This research reiterates the importance of a clear understanding of the purpose, objectives and rationale for change (i.e., discrepancy and appropriateness) in developing readiness. Middle managers and frontline providers could be invited to participate in planning the change, as this allows discussions surrounding widespread benefits of change – such as the individual, patient, and health system, which help enhance valence. Inclusion of employees in planning would also bolster understandings of the change process and perceptions of procedural fairness. Attention also needs to be paid to distributive fairness when decisions and communications are made on resource allocation and distribution. This is particularly important when the change is to be implemented across sites that are geographically dispersed. With respect to training – an important element of most organizational change – this study points to the importance of ongoing learning, informal training and training on the *process* (in addition the content) of change as both can contribute to an individual’s confidence in their ability to undertake the change. The study also shows the need for senior managers to attend to middle management readiness for change. Middle managers need to be equipped with adequate information on the change and the needed skills to exude understanding and confidence to the frontline providers. If middle managers themselves are not ready for change, it will be difficult for them to create readiness among frontline employees.

There is no doubt that change is ubiquitous in health care organizations, prompting managers and employees at all levels to engage with it. Understanding individual readiness should be part of every health care organization member’s toolbox.

## Supplementary information

**Additional file 1.** Interview Guide

## Data Availability

We have included the interview guide in Additional file 1. Interview transcripts and meeting observation notes are not available to preserve the anonymity of participants.
