# Supplementary material for: Examining health care providers’ and middle-level managers’ readiness for change: a qualitative study
Source: BMC Health Serv Res. 2020 Jan 17;20:47. doi: 10.1186/s12913-020-4897-0 (PMC6969476; doi:10.1186/s12913-020-4897-0)
Supplement: Supplementary file 1 — Additional file 1. Interview Guide [file 12913_2020_4897_MOESM1_ESM.docx]

**Additional file 1: Interview Guide**

CONTEXT

Can you tell me about your role in the [Senior Care Program]. What are your responsibilities?

Please tell me about the [SCP].

What are the objectives of the program?

In your opinion, is the program meeting these objectives?

Could you describe Health Links to me?

How does the process of care coordination work?

From your perspective, what are the similarities between Health Links and [SCP]? (Similarities in terms of the process of care coordination? Objectives?)

In contrast, how are the programs different?

In your opinion, what is the objective of integrating Health Links with the [SCP]?

Can the integration of the two successfully meet these objectives? If not, why so?

How have your role, responsibilities, and workload changed in preparation for Health Links?

Have you been asked to select patients from your roster to complete a CCP?

Do you have a target number of CCPs that you must complete?

Prior to the integration of Health Links and the [SCP], what planning took place?

How did you first hear about Health Links and that you would be required to work in the approach?

READINESS

What is your view of the integration?

Is there a need to integrate Health Links with SCP? Why or why not?

How does the integration align with the CHC’s mission and values?

How was the integration communicated to you? (What was communicated and how?)

Can you please elaborate on whether and how this helped to foster a sense of necessity for this specific change?

Do you believe that the integration should be a priority? Why or why not?

What value or benefits do you see in integrating Health Links and [SCP]?

How does the integration affect your workload?

How does the integration of Health Links change the current process of health service delivery for you?

How do you feel about participating in a Health Link?

How have your professional experiences and training influenced your readiness?

What are your opinions on your capability to carry out the changes in your responsibilities that will result from the integration?

How did you prepare for the integration?

Were there shadow visits? Webinars? Meetings with LHIN/Health Links representatives?

What training took place? (e.g. training on completing CCP? Training on CHRIS?

What is your view of the training? (Was it useful?)

What other resources were provided to facilitate the integration?

Were these resources distributed fairly across the sites?

How were decisions made about the integration at your site?

Were you consulted about the changes? If so, how?

Was there anything that could be done to make this process easier for you?

What else could have been done to make you feel more ready for the integration?

LEADERSHIP

How would you describe the leadership of the integration of Health Links and [SCP]?

What leadership has been provided? Who is it from?

What are the expectations from leadership with respect to the implementing the HL approach?

Do your expectations align with expectations of those leading the change?

How would you describe the leadership style?

Would you say the leadership within your CHC is different from the leadership from higher levels?

Is leadership consistent across sites?

Is there anything you would suggest be changed regarding how the integration is being lead?

CHALLENGES AND ENABLERS

What are other challenges associated with the integration of Health Links and the [SCP] that we have not discussed?

How can these challenges be overcome?

What are the facilitators of the integration that we have not yet discussed?

How do these factors act as facilitators?

If you could change anything about the preparation process of the integration of Health Links and [SCP], what would you change?

CONCLUDING QUESTIONS

Are there any other experiences you would like to share with me to help me better understand your readiness for the integration of Health Links and [SCP]?

Is there anything I did not ask you during the interview that I should have asked?

Is there anyone else who you think I should speak to and who would be willing to speak with me as part of this study?

Would you be willing to forward the recruitment email to other individuals involved with Health Links and [SCP] who may be interested in participating in this study?
